# Non-Contact Measurement of the Spectral Emissivity through Active/Passive Synergy of CO_2_ Laser at 10.6 µm and 102F FTIR (Fourier Transform Infrared) Spectrometer

**DOI:** 10.3390/s16070970

**Published:** 2016-06-24

**Authors:** Ren-Hua Zhang, Hong-Bo Su, Jing Tian, Su-Juan Mi, Zhao-Liang Li

**Affiliations:** 1 Key Laboratory of Water Cycle and Related Land Surface Processes, Institute of Geographical Sciences and Natural Resources Research, Chinese Academy of Sciences, Beijing 100101, China; zhangrh@igsnrr.ac.cn (R.Z.); misj.12b@igsnrr.ac.cn (S.M.); 2 Department of Civil, Environmental and Geomatics Engineering, Florida Atlantic University, Boca Raton, FL 33431, USA; hongbo@ieee.org; 3 University of Chinese Academy of Sciences, Beijing 100049, China; 4 Key Laboratory of Agri-informatics, Ministry of Agriculture/Institute of Agricultural Resources and Regional Planning, Chinese Academy of Agricultural Sciences, Beijing 100081, China; lizhaoliang@caas.cn; 5 ICube, UdS, CNRS, 300 Boulevard Sebastien Brant, CS 10413, Illkirch 67412, France

**Keywords:** 10.6 µm single-band CO_2_ laser, temperature-emissivity retrieval, active/passive synergistic measurement and inversion

## Abstract

In the inversion of land surface temperature (LST) from satellite data, obtaining the information on land surface emissivity is most challenging. How to solve both the emissivity and the LST from the underdetermined equations for thermal infrared radiation is a hot research topic related to quantitative thermal infrared remote sensing. The academic research and practical applications based on the temperature-emissivity retrieval algorithms show that directly measuring the emissivity of objects at a fixed thermal infrared waveband is an important way to close the underdetermined equations for thermal infrared radiation. Based on the prior research results of both the authors and others, this paper proposes a new approach of obtaining the spectral emissivity of the object at 8–14 µm with a single-band CO_2_ laser at 10.6 µm and a 102F FTIR spectrometer. Through experiments, the spectral emissivity of several key samples, including aluminum plate, iron plate, copper plate, marble plate, rubber sheet, and paper board, at 8–14 µm is obtained, and the measured data are basically consistent with the hemispherical emissivity measurement by a Nicolet iS10 FTIR spectrometer for the same objects. For the rough surface of materials, such as marble and rusty iron, the RMSE of emissivity is below 0.05. The differences in the field of view angle and in the measuring direction between the Nicolet FTIR method and the method proposed in the paper, and the heterogeneity in the degree of oxidation, polishing and composition of the samples, are the main reasons for the differences of the emissivities between the two methods.

## 1. Introduction

Large-scale information on land surface temperature (LST) is of great importance to global changes, water circulation and balance, crop production forecast, agricultural drought monitoring, urban heat islands, and some other fields. In the inversion of land surface temperature with airborne and satellite data, gaining information on land surface emissivity is most challenging.

By far, humans can only roughly obtain satellite pixel scale emissivity through temperature-emissivity retrieval algorithms, which are related to atmospheric radiation and transmission, rather than directly measuring land surface emissivity using the airborne or satellite platforms.

Temperature-emissivity retrieval is challenging because both the true land surface temperature and the emissivity contained in the thermal infrared radiation signal measured at a certain waveband are unknown, which makes the radiative transfer equations not closed. As only the infrared radiation information (radiation temperature) measured by the sensor is available, the number of equations increases with that of the wavebands, leading to n + 1 unknowns in n equations. Therefore, it is impossible to directly solve the underdetermined equations without any additional condition. Either removing one of the unknowns, or inverting both emissivity and temperature, is the goal of separating temperature and emissivity.

At the end of the 1970s, scientists in quantitative remote sensing paid great attention to temperature-emissivity retrieval algorithms and proposed multiple methods to obtain information about land surface emissivity and, thus, to invert LST. Over the past three decades, all of the methods, including Kahle’s reference channel method [1], Watson’s two-temperature method and spectrum ratio method [2,3], Gillespie’s normalization MMD method [4], alpha-derived emissivity method [5,6], Becker and Li’s day/night temperature independent spectral indices (TISI)-based methods [7,8,9,10,11,12], Wan and Li’s physics-based day/night operational methods (D/N) [13,14], Barducci’s gray body emissivity methods (GBE) [15], Gillespie’s temperature emissivity separation methods (TES) [4,16,17,18], Borel’s iterative spectrally smooth TES methods (ISSTES) [19,20,21], Wang et al.’s separation methods (LECTES) [22], Aires et al.’s artificial neural network methods (ANN) [23,24,25], and Lietal and Ma’s two-step physical retrieval methods (TSRM) [26,27,28], have focused on removing one of the unknowns with prior knowledge, as well as by introducing certain assumptions and statistical methods. For example, the temperature-independent spectral indices method, which was proposed by Becker and Li [7,8,9], uses directly measured data in the daytime and the nighttime within the mid-infrared and thermal infrared wavebands based on temperature-independent spectral indices to obtain the thermal infrared emissivity. That method removes one of the unknowns and realizes the separation between temperature and emissivity. Inspired by the day/night TISI based method, Wan and Li [13] further developed a physics-based D/N method to simultaneously retrieve LST and emissivity from a combined use of the day/night pairs of middle-infrared and thermal-infrared data. This method assumes that surface emissivity does not significantly change from day to night, and that the angular form factor has very small variations (<2%) in the middle-infrared wavelength, which reduce the number of unknowns and make the retrieval more stable. Due to its good performance, the method was chosen by NASA to derive the land surface temperature data product from MODIS [13]. Krishnan et al. compared in situ, aircraft, and satellite land surface temperatures and the results showed that the standard deviation of the brightness temperature during the flight periods were <1.7 °C [29]. Moreover, there are generalized single-channel algorithms for retrieving land surface temperature from remote sensing data, in which emissivity is obtained by using reflectivity and vegetation indices [30,31].

In addition to these temperature-emissivity retrieval methods, is there possibility of direct measuring emissivity from space? If so, the ill-conditioned equations in the retrieval of LST and emissivity with satellite data will be resolved and the retrieval of LST and emissivity will become much easier. So far, a number of methods for emissivity field measurement have been presented, such as the box method [32,33], the Fourier transform method [34], and the CO_2_ laser method. The CO_2_ laser method, which uses an active thermal infrared radiation source, is shown as the most promising way of measuring emissivity from space [35,36,37,38] because CO_2_ lasers can emit strong thermal infrared radiation from the airborne or satellite platforms, changing the land surface thermal infrared irradiance and, thus, obtaining land surface emissivity within the CO_2_ laser waveband.

In 1991, the Scientific Research Center of the University of Strasbourg studied the polarization scattering coefficient of CO_2_ laser scattered by soil, and validated the model with directional emissivity data [39]. In 1996, the same research group conducted further research by measuring the emissivity of bare soil using the scattered CO_2_ laser, and made in-depth analysis of the relations between absolute calibration and backscattering [40]. As the infrared radiometers used in the above experiments work at a wavelength of 8–14 µm, the corresponding waveband differed greatly from the waveband of the CO_2_ laser, and the emissivity measurement corresponds to an averaged energy within 8–14 µm from the CO_2_ laser source of 10.6 ± 0.15 µm [41]. Since the wide waveband emissivity averages the variability in the 8–14 µm thermal infrared spectrum, many applications that require detailed spectral emissivity changes are restricted. In addition, it is generally assumed that only a CO_2_ laser instrument with a spectral distribution of 8–14 µm can be used to close the ill-conditioned equations in the retrieval of LST with satellite data. In fact, however, a CO_2_ laser instrument with a narrow band, such as 10.6 µm used in the study, can be used to retrieve not only LST, but also emissivity, with a spectral distribution of 8–14 µm, which is a main idea of this paper. The main difference between the method proposed in the paper and the previous CO_2_ laser measurement technique is that this method is developed to measure the spectrum of the surface object with the combination of CO_2_ laser and the 102F portable FTIR instrument, while the previous studies can only measure the emissivity in a single narrow wavelength which is the same with the wavelength of the CO_2_ laser.

In the study, a method of measuring the emissivity spectrum in 8–14 µm with the CO_2_ laser at 10.6 µm ± 0.15 µm and the 102F FTIR spectrometer is proposed in the paper. Section 2 introduces the methodology and Section 3 describes the major instruments used in the experiments. Section 4 presents the experimental results and data analysis. The last section gives the conclusions and discussion.

## 2. Methodology

### 2.1. Measuring Concepts

In the method of using the active/passive synergy of a CO_2_ laser source to measure the spectral distribution of land surface emissivity, the radiative balance equation requires not only a thermal infrared spectrometer but also a CO_2_ laser source. In the study, the spectral distribution of land surface emissivity within the 8–14 µm range is obtained by using the 10.6 µm single-band CO_2_ laser and a 102F FTIR spectrometer (D&P Instruments, SIMSBURY, CT, USA).

**Figure 1:**
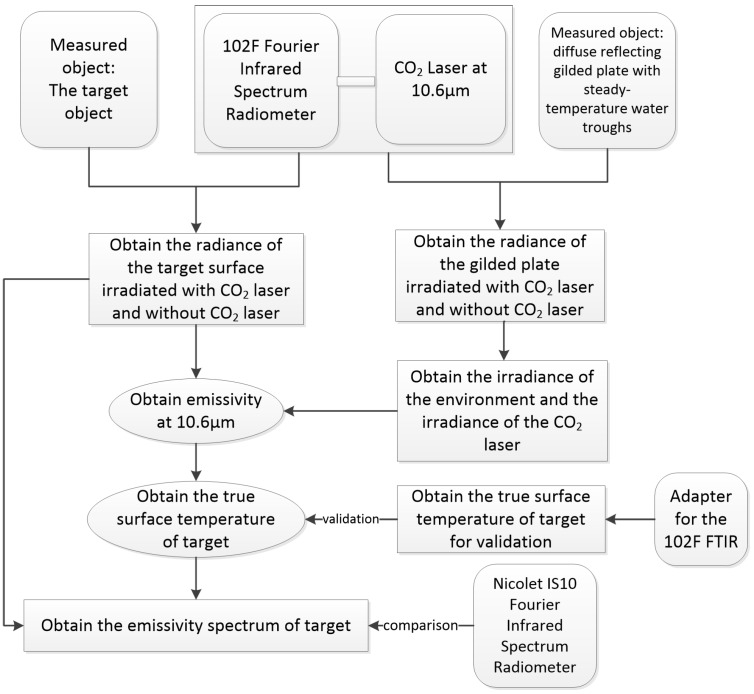
Flowchart of retrieving the emissivity spectrum of the target surface.

Figure 1 shows the frame of retrieving the emissivity spectrum of the target surface.

The concept for measuring is as follows: firstly, we need to determine the irradiance of CO_2_ laser beams at 10.6 µm with the steady-temperature diffusing gilded plate, and then measure the radiance of the target surface with an irradiating laser, and without the irradiating laser, at 10.6 µm with the 102F FTIR spectrometer measurement. After obtaining the irradiance of the CO_2_ laser and the radiance of the target surface at 10.6 µm, we can calculate the emissivity at the 10.6 µm waveband according to the radiance equations with an irradiating laser, and without the irradiating laser. That helps to close the underdetermined equations at the 10.6 µm waveband and invert the true temperature of the target surface, with which we can close the underdetermined equations of the N channels and finally invert the emissivity of the N channels. That is to say, the land surface emissivity and true temperature of each waveband in the spectrum measured in synergetic inversion are solved. The following section describes the measuring procedures in detail.

### 2.2. Measuring Procedures and Algorithm Derivation

#### 2.2.1. The Determination of the Emissivity at 10.6 µm ($\varepsilon_{10.6\mu }$)

In the 10.6 ± 0.15 µm waveband, the radiation equation for the measured object without a CO_2_ laser is as Equation (1) and the radiation equation for the measured object surface irradiated by the CO_2_ laser at the 10.6 µm waveband is as Equation (2):

(1) $$R_{e10.6\mu }=\varepsilon_{10.6\mu }\;B_{10.6\mu }+(1-\varepsilon_{10.6\mu })L_{e10.6\mu }$$

(2) $$R_{eJ10.6\mu }=\varepsilon_{10.6\mu }\;B_{10.6\mu }+(1-\varepsilon_{10.6\mu })(L_{e10.6\mu }+L_{J10.6\mu })$$

where $R_{eJ10.6\mu }$ and $R_{e10.6\mu }$ is the radiance with, and without, the irradiation of the CO_2_ laser measured by the sensor. $B_{10.6\mu }$, $L_{e10.6\mu }$, $L_{J10.6\mu }$, and $\varepsilon_{10.6\mu }$ are, respectively, the black-body radiance of the sample, the irradiance of the environment, the irradiance of the CO_2_ laser, and the emissivity of the sample at the 10.6 µm wavelength. In case of a quick shift (several seconds) between laser irradiation and non-irradiation, during the response time of sensors, measured objects can be viewed as having a constant temperature. Equation (2) minus Equation (1) gives:

(3) $$\varepsilon_{10.6\mu }=1-\;\frac{R_{eJ10.6\mu }-R_{e10.6\mu }}{L_{J10.6\mu }}$$

According to Equation (3), $\varepsilon_{10.6\mu }$ can be estimated from $R_{eJ10.6\mu }$, $R_{e10.6\mu }$ and $L_{J10.6\mu }$. As mentioned, $R_{eJ10.6\mu }$ and $R_{e10.6\mu }$ are measured directly by the 102F FTIR. Therefore, as long as $L_{J10.6\mu }$ is determined, $\varepsilon_{10.6\mu }$ is obtained.

#### 2.2.2. The Determination of the Irradiance of the CO_2_ Laser ($L_{J10.6\mu }$)

Using a diffuse reflecting gilded plate as the sample whose emissivity is known, $L_{J10.6\mu }$ is determined.

Similar with Equation (1), the radiation equations with and without the irradiation of the CO_2_ laser are expressed as Equations (4) and (5), respectively:

(4) $$R_{ge10.6\mu }=\varepsilon_{g10.6\mu }\;B_{g10.6\mu }+(1-\varepsilon_{g10.6\mu })L_{e10.6\mu }$$

(5) $$R_{geJ10.6\mu }=\varepsilon_{g10.6\mu }\;B_{g10.6\mu }+(1-\varepsilon_{g10.6\mu })(L_{e10.6\mu }+L_{J10.6\mu })$$

where the subscript g denotes the diffuse reflecting gilded plate. Other parameters have the same meanings as that in Equations (1) and (2). $L_{J10.6\mu }$ is derived using Equation (5) minus Equation (4):

(6) $$L_{J10.6\mu }=\;\frac{R_{geJ10.6\mu }-R_{ge10.6\mu }}{1-\varepsilon_{g10.6\mu }}$$

$R_{ge10.6\mu }$ and $R_{geJ10.6\mu }$ are directly measured by the 102F FTIR. $\varepsilon_{g10.6\mu }$ is known as the emissivity of the diffuse reflecting gilded plate. In this way, $L_{J10.6\mu }$ can be obtained and then is substituted into Equation (3), $\varepsilon_{10.6\mu }$ is calculated.

#### 2.2.3. The Determination of the Irradiance of the Environment ($L_{e10.6\mu }$)

To obtain the true temperature of target surface in terms of Equation (3), $L_{e10.6\mu }$ still need to be determined. In this study, to reduce the effects of the temperature change of the gilded plate on the measurement, a diffuse reflecting gilded plate with steady-temperature water troughs is designed to keep the temperature of the gilded plate constant. During the measurement, the trough is filled with water and a six-point thermocouple thermometer is tightly embedded to measure the surface temperature. Details about this device are described in Section 3.3. In this way, the black-body radiance of the gilded plate ($B_{g10.6\mu }$) can be determined according to the Planck’s equation (see Equation (7)) and, thereby, $L_{e10.6\mu }$ can be calculated according to Equation (4).

(7) $$B(T,\lambda )=\frac{C_{1}\lambda^{-5}}{\exp(\frac{C_{2}}{\lambda T})-1}$$

where B(T,λ) is the black-body radiation at any waveband (λ) (*C*_1_ = 3.74 × 10^8^ W·µm^4^·m^−2^, *C*_2_ = 1.439 × 10^4^ µm·K). *T* is the black-body temperature.

#### 2.2.4. The Calculation of the True Temperature of the Target Surface (*Ts*)

Combining Equations (6) and (3), $\varepsilon_{10.6\mu }$ is calculated. Then putting $\varepsilon_{10.6\mu }$, $L_{e10.6\mu }$ into Equation (1), $B_{10.6\mu }$ can be computed and thereby the true temperature of the sample is obtained according to Equation (7). In the study, an adapter for the 102F FTIR is developed to measure *Ts* and to validate the retrievals of *Ts* further, which is described in Section 3.4.

#### 2.2.5. The Determination of the Emissivity Spectrum of the Target Surface

After the determination of *Ts*, the emissivity spectrum can be obtained easily on the basis of the radiation equation.

Similar with Equation (1), the radiance in all wavelengths (*R*) measured by the 102F FTIR is as Equation (8):

(8) $$R=\varepsilon_{s}B_{s}+\left(1-\varepsilon_{s}\right)L_{e}$$

where $\varepsilon_{s}$ is the emissivity of the target surface at all wavebands, *Bs* is the black body radiation of the target surface, and *Le* is the irradiance of the environment.

The same method described in Section 2.2.3 is used to determine *Le*. The blackbody radiance at all wavelengths is calculated from *Ts* according to Planck’s equation. Putting *R*, *Bs*, and *Le* into Equation (8), the emissivity spectrum of the sample is calculated.

To evaluate the method of retrieving the true temperature of the sample (see Section 2.2.4), an adapter of the 102F FTIR spectrometer is used in this study to measure the true temperature, which is described in Section 3.4, and to validate the retrieved emissivity spectrum, the emissivity spectrum measured with the Nicolet IS10 FTIR spectrometer (Thermo Fisher Scientific, Waltham, MA, USA) is compared with that retrieved by the method above.

## 3. Measuring Instruments

In the experiment, six instruments or devices are used.

### 3.1. 102F FTIR Spectrometer

The 102F FTIR spectrometer is designed and manufactured by the D&P Instrument, SIMSBURY, CT, USA), which is used to measure the total radiance including the radiance emitted by the sample object and the radiance from the environment reflected by the sample. Its main specifications are listed in Table 1.

### 3.2. The CO_2_ Laser

The CO_2_ laser used in the study is a C-20A RF-excited waveguide sealed CO_2_ laser produced by the Coherent, Inc (Santa Clara, CA, USA). The air-based cooling system is light and highly stable.

**Figure 2:**
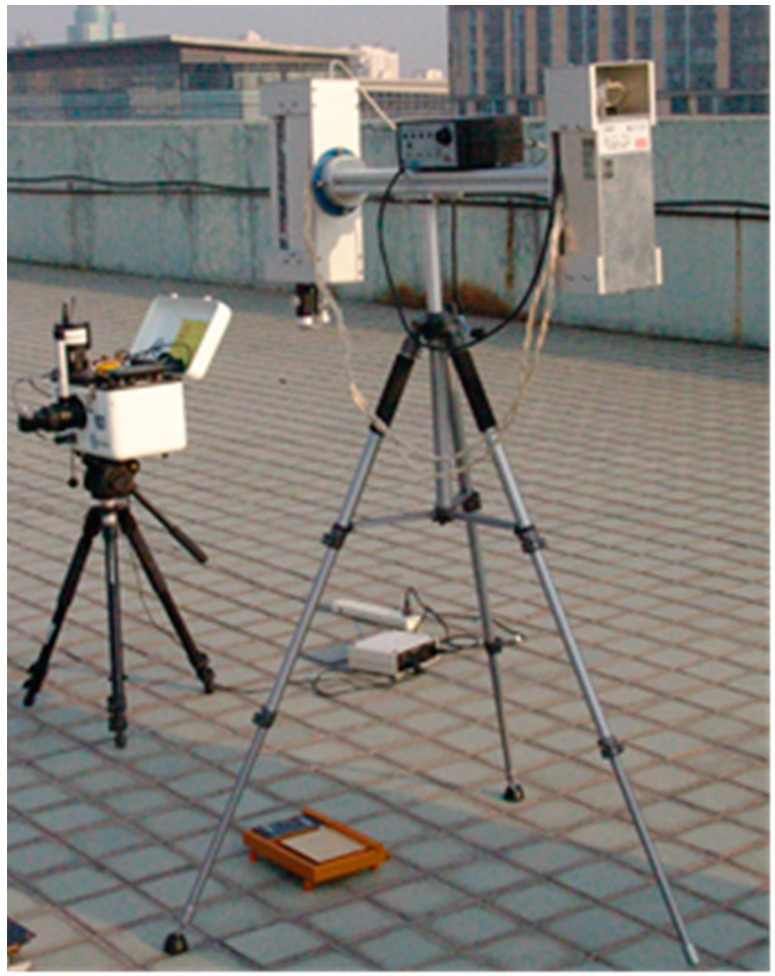
Air-cooling 10.6 µm CO_2_ laser source and the 102F FTIR spectrometer.

Technical parameters of the CO_2_ laser are provided in Table 2. Figure 2 shows the photo of the CO_2_ laser, the 102F FTIR spectrometer, and the diffuse reflecting gilded plate.

### 3.3. Diffuse Reflecting Gilded Plate with Steady-Temperature Water Troughs

**Figure 3:**
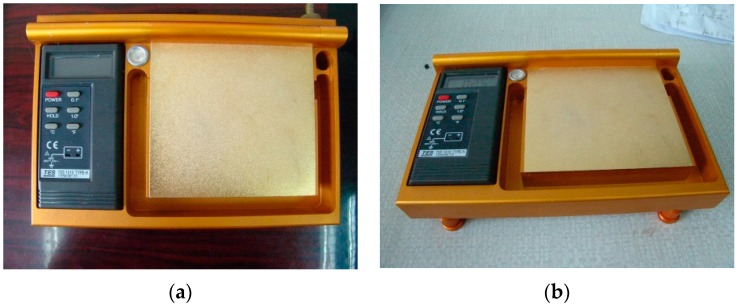
Photos of the diffuse reflecting gilded plate with steady-temperature water troughs.

Figure 3 shows the multi-point steady-temperature gilded plate developed by the authors, which consists of a gilded plate, multi-point thermocouple point-thermometers and steady-temperature water troughs. Six thermocouple point-thermometers are tightly embedded and evenly distributed on the back of the gilded plate, and are soaked in steady-temperature water troughs. Moreover, they are connected in parallel to the data logger. On the plate surface, there is also a level device to adjust the horizontal level of the steady-temperature water troughs.

Plenty of water in the steady-temperature water troughs can ensure the consistency between the temperatures measured by the six thermocouple point-thermometers soaked in the troughs. Moreover, the large thermal inertia of water can ensure the stability and spatial representativeness of the temperature measured by the six thermocouple point-thermometers with the passage of time. The average of the six temperatures is used as the temperature of the gilded plate. This device was approved by China’s patent authority as an invention patent in 2014, with a patent number as ZL201210362463.

### 3.4. The Adapter of the 102F FTIR Spectrometer

In the application of the 102F spectrometer, it is assumed that the sample has a maximum emissivity close to 1 at a certain waveband and thereby the black-body radiation at this waveband can be used to simulate and calculate the true temperature of the measured object [42]. Obviously, this assumption only suits for the samples with high emissivities, such as 0.98–0.99, while for minerals or metals whose emissivity are usually low, the errors of the true temperature of the sample may not be acceptable.

**Figure 4:**
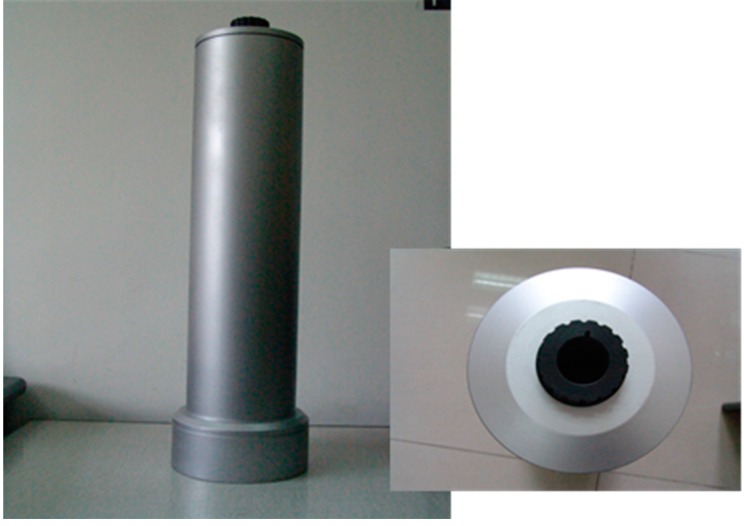
Photos of the adapter of 102F FTIR spectrometer taken, respectively, from the side and from above.

To measure the true temperature of the sample, an adapter for 102F FTIR spectrometer (which was certified by China’s patent authority as an invention patent in 2014, with the patent number as ZL201210363506.X) is designed by the authors, which is shown in Figure 4.

In the measurement, the sample is covered by the adapter. Through the hole, with the same size as the lens of the 102F FTIR, on the top of the adapter, the radiance is measured. Equation (9) gives the radiation equation:

(9) $$R=\varepsilon_{s}B_{s}+\left(1-\varepsilon_{s}\right)E$$

where the subscript s represents the sample, and *E* represents the irradiance of the adapter. When *R* = *E*, meaning the radiation temperature of the inner surface of the adapter equals to the radiation temperature of the sample, Equation (10) is rewritten as:

(10) $$\varepsilon_{s}B_{s}+\left(1-\varepsilon_{s}\right)R-R=0$$

From Equation (10), $B_{s}=R$ is derived, which illustrates that the measured radiance through the adapter is equal to the black body radiation of the sample. According to the Planck’s equation, the true temperature of the sample can be retrieved.

This method requires the radiation temperature of the inner surface of the adapter equals to the radiation temperature of the sample. In the study, the inner surface of the adapter of the 102F FTIR spectrometer is specially treated with black paint whose emissivity is close to 1.0. When the sample and the adapter are in the same environment for enough time, their radiation temperatures are almost the same. During the measurement, the radiation temperature of the inner surface of the adapter and the radiation temperature of the sample are observed with the infrared radiation thermometer. When they are equal, the radiance of the sample covered by the adapter is observed and is used to retrieve the true temperature of the sample. This measured true temperature is then used to be compared with the temperature obtained with the method in Section 2.2.4.

### 3.5. Nicolet IS10 FTIR Spectrometer

A Nicolet IS10 FTIR spectrometer with an integrating sphere can measure the directional-hemispherical reflectance of the object directly and directional-hemispherical emissivity is calculated from the reflectance according to the Kirchhoff’s law. This instrument is usually used indoors and can only measure the sample with a diameter of about 2 cm. Due to the differences in the field of view angle and in the measuring direction between the 102F FTIR and the Nicolet FTIR, the emissivity measured by the two instruments must exist difference. However, because the Nicolet FTIR method is regarded as the most accurate in the measurement of surface emissivity [43], it is still used to evaluate the proposed method in the paper.

## 4. Experiment and Validation

### 4.1. Experiment Description

The experiment was conducted on the roof experimental field of the building of the Institute of Geographic Sciences and Natural Resources Research, Chinese Academy of Sciences in September 2011, in which several typical objects were tested according to the method discussed in this paper using the air-cooling 10.6 µm CO_2_ laser source, 102F FTIR spectrometer (see Figure 2), and diffuse reflecting gilded plate with steady-temperature water troughs (see Figure 3).

The typical objects include aluminum plate, iron plate, copper plate, marble plate, rubber sheet, and paper board. During the measurement, there were no clouds in the sky and the ambient (including the sky, instruments, and observers) downward thermal infrared radiation was steady. The beam from the CO_2_ laser vertically falls on the central part of the measured objects. The light spot of the laser beam had a diameter of about 10 cm. There is a shifting device in front of the silicon lens that can make the laser beam irradiate or not irradiate the measured objects. During the observation, observers strictly remained at the fixed location to avoid influence on the stability of the downward thermal infrared radiation. Observation was advanced in accordance with the measuring steps in Section 2.2.

### 4.2. Validation of the Retrievals of the True Temperature of the Sample

Using the method described in Section 2.2, the emissivities at 10.6 µm are first retrieved, and then the true temperatures of the samples are calculated. Table 3 shows the emissivities of the six samples at 10.6 µm, with which the true temperatures are calculated using the method proposed in Section 2.2.4.

To avoid confusion, the retrieved temperature based on the method above is defined as *T_S_e_* and the temperature measurement with the adaptor is defined as *T_S_m_*.

Table 3 shows that the estimates of *Ts* (*T_S_e_*) are very close to the measurements. The largest difference between *T_S_m_* and *T_S_e_* is 0.9 K. This result illustrates that the method of retrieving the true temperature of the sample proposed in Section 2.2 is reliable.

### 4.3. The Comparison of the Emissivities Retrieved by the Nicolet FTIR Spectrometer and by the CO_2_ Laser and 102F FTIR Spectrometer

**Figure 5:**
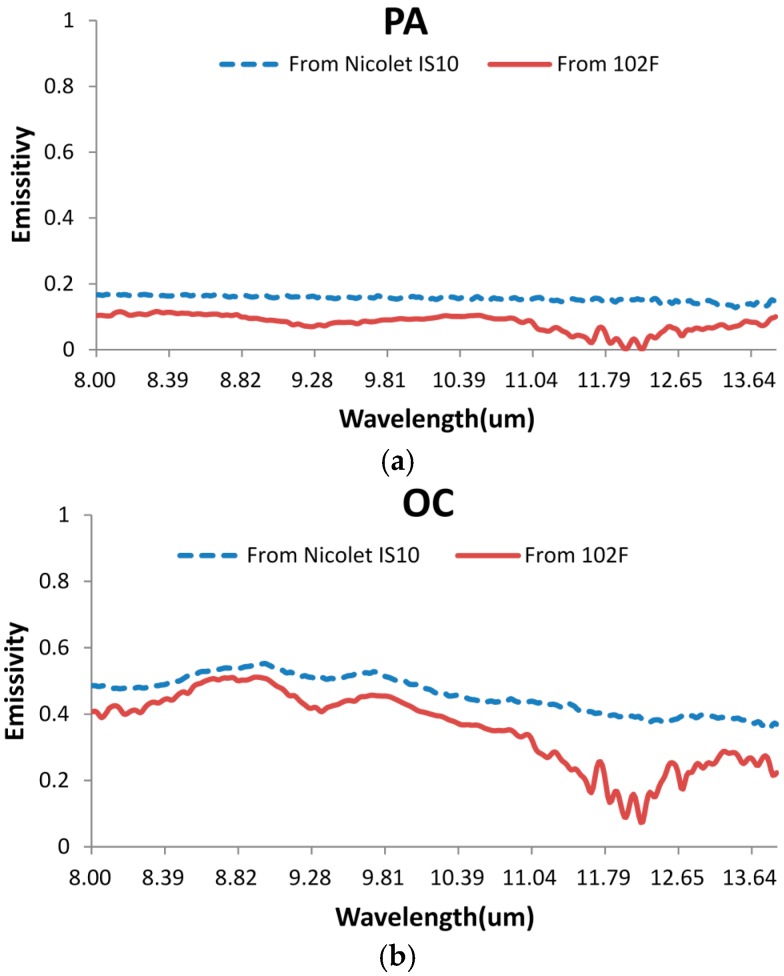

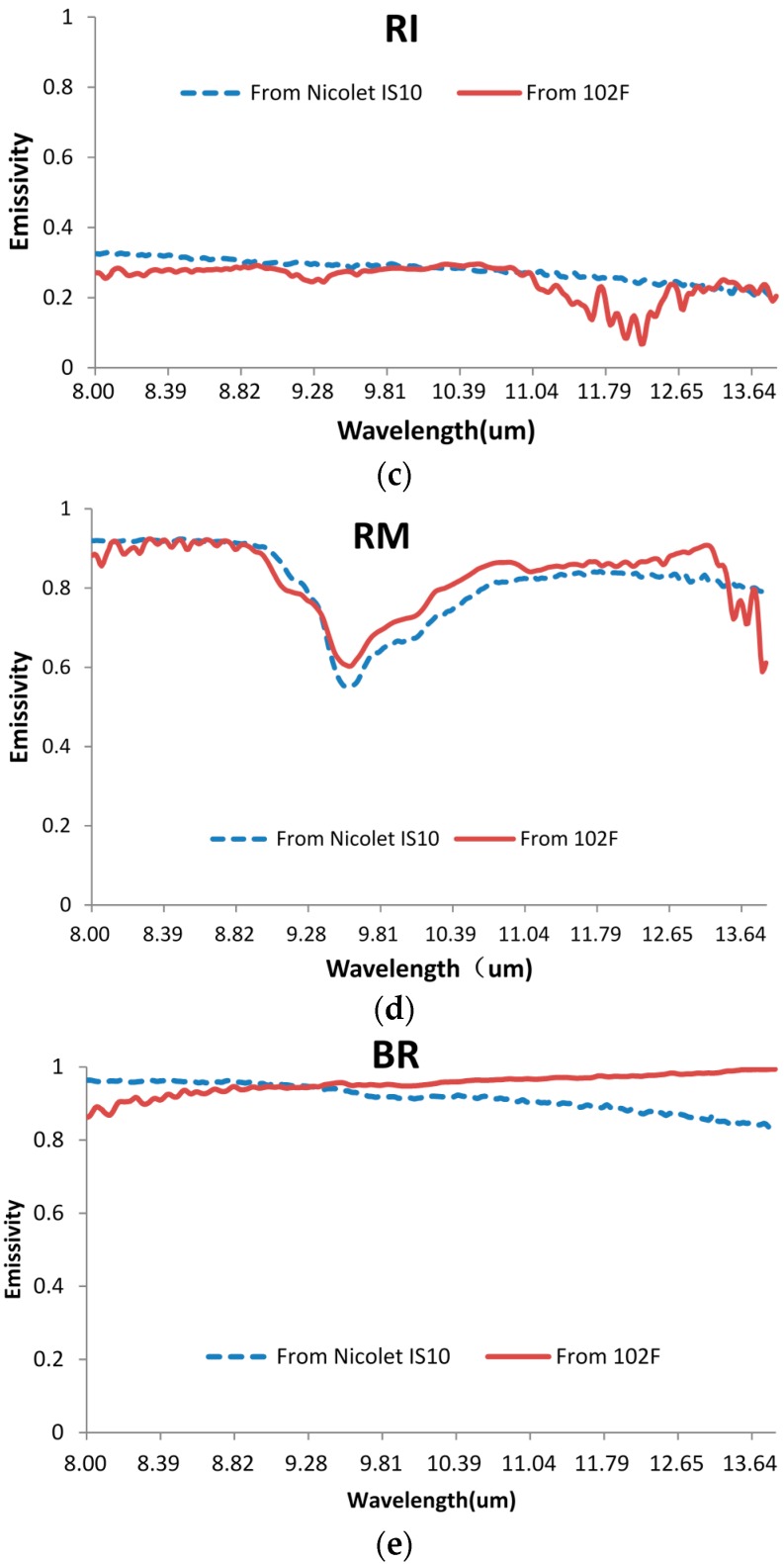
(**a**) Comparison of the emissivity spectral distribution of polished aluminum plate measured with two different instruments at 8–14 µm; (**b**) comparison of the emissivity spectral distribution of copper plate measured with two different instruments at 8–14 µm; (**c**) comparison of the emissivity spectral distribution of iron plate measured with two different instruments at 8–14 µm; (**d**) comparison of the emissivity spectral distribution of marble plate (rough surface) measured with two different instruments at 8–14 µm; and (**e**) comparison of the emissivity spectral distribution of rubber sheet measured with two different instruments at 8–14 µm.

To evaluate the method of measuring emissivity proposed in the paper, emissivities of the five objects (RM, PA, OC, RI, and BR) were measured by the Nicolet FTIR spectrometer with the integrating sphere and by the CO_2_ laser and 102F FTIR spectrometer. The comparisons between the two sets of results are given (see Figure 5a–e). The RMSE of the spectral emissivity from the two approaches is provided in Table 4.

The comparison shows the following facts:

The magnitude level and the spectral distribution of the emissivity at 8–14 µm measured with the two approaches are basically the same, and the results measured with the method described in the paper are slightly lower.

As for the objects with rougher surfaces, such as the rough surface of marble plate, the two results are more consistent, with RMSE less than 0.05.

As for the objects with smoother surfaces, the two results are less consistent, and the emissivity spectrum shows more peaks at 11–14 µm when measured with the method described in the paper than that measured with the Nicolet IS10 method.

The major reasons for the difference of the emissivity spectrum between the two approaches lie in their differences in the field of view angle and in the measuring direction.

Emissivity from the Nicolet IS10 FTIR spectrometer with the integrating sphere is directional-hemispherical, while the emissivity from the CO_2_ laser and the 102F spectrometer is directional-directional. This implies that the view angle is quite different for the two instruments. For the materials with a good isotropy of emissivity, the directional-directional emissivity and the directional-hemispherical emissivity will be very close to each other for the same material, since the emissivity does not vary with the view angles. However, the samples used in the experiment are not isotropic, due to the heterogeneity in the degree of oxidation, polishing, and composition. As a result, this difference between the two approaches would produce some differences between the measurements of the emissivity of the samples. The larger deviation of the emissivity for the smooth surface may be caused by the difference of the view angle between the two instruments because directional emissivity changes abruptly with a slight change of view angle from those smooth surfaces. In addition, 102F portable FTIR instrument is for outdoor use, while the Nicolet FTIR is used indoors. Any change of the surrounding environment, such as the observer’s position or sky condition, could affect the measurement of the 102F and lead to the fluctuations of the spectrum.

The spectral distribution of emissivity is very sensitive to the composition and surface roughness of the sample, which is challenging to the validation of the measured results. Since the Nicolet FTIR method is the only precise way of validating the emissivity measurement it is used, although there is a difference in the field of view angle and in the measuring direction between it and the method proposed in the paper.

## 5. Conclusions and Discussions

### 5.1. Conclusions

In this paper, a method of measuring the spectral distribution of land surface emissivity by using the 10.6 µ single-band CO_2_ laser and the 102F FTIR spectrometer is proposed. Firstly, the irradiance of the CO_2_ laser is measured with the multi-point steady-temperature gilded plate and the 102F FTIR spectrometer. The advantage of the multi-point steady-temperature gilded plate is that it can ensure the stability and spatial representativeness of the temperature of the gilded plate through using the water trough, which avoids the effects of the temperature change of the gilded plate on the measurement. Secondly, the emissivity of the sample at the 10.6 µm narrow waveband is obtained and is then used to invert the true temperature of the sample according to the radiation equation. Thirdly, substituting the retrieved true temperature of the sample into the radiation equation, the underdetermined equation of the N channel is closed and the emissivity of the N channel is finally inverted.

The emissivities of several materials, including aluminum plate, iron plate, copper plate, marble plate, rubber sheet, and paper board are measured in the study. To validate the inverted true temperature of the samples, an adapter for the 102F FTIR spectrometer is specially developed to directly measure the true temperature of the sample. Its measuring principle is that when the radiation temperature of the environment equals the radiation temperature of the sample, the radiance of the sample measured in this environment is its black body radiation, from which the true temperature of the sample can be calculated. The result of the validation shows that it is very close to each other between the inverted true temperature and the measurements.

By comparison with the emissivity measurement from the Nicolet IS10 FTIR spectrometer, the method of measuring emissivity presented in the paper is evaluated. The results show that the magnitude level and the spectral distribution of the emissivity at 8–14 µm measured with the two approaches are basically the same, and the emissivities measured with the method described in this paper are slightly lower. The RMSE of the emissivities for the objects with rougher surface (less than 0.05) are smaller than that for the objects with smooth surface. The differences in the field of view angle and in the measuring direction between the Nicolet FTIR method and the method proposed in the paper, and the heterogeneity in the degree of oxidation, polishing, and composition of the samples are the main reasons for the differences of the emissivities between the two methods.

### 5.2. Discussion

By adding an active thermal radiation source, such as a CO_2_ laser, land surface emissivity can be measured using the method presented in the paper. It helps close the underdetermined equations for the thermal infrared radiation of measured objects. The additional information on emissivity will make a breakthrough in the algorithms of temperature-emissivity retrieval and lead to a new approach for inverting land surface temperature. The more important thing is that this method provides an idea for the direct measurement of emissivity remotely from the airborne or spaceborne platform. If it is possible to add a CO_2_ laser from the airborne platform onto the surface, land surface emissivity probably could be achieved with the method proposed in the paper, which will have a significant impact on the remote sensing and geoscience community. Certainly, it is just a vision for the future and has a long way to go.

## Figures and Tables

**Table 1:** Specifications of the 102F FTIR spectrometer.

Name	Characteristics
Spectral range	2 to 16 mircon meter
Spectral resolution	4 cm^−1^, 8 cm^−1^, and 16 cm^−1^ (adjustable), 2 cm^−1^ can be chosen
Spectral precision	±1 cm^−1^ covers the whole spectral range
Scanning speed	1 scan/s, 4 cm^−1^ resolution
Sampling frequency	5 kHz
Signal bandwidth	2 kHz

**Table 2:** The characteristics of the CO_2_ laser used in the study.

Type: C-20A	Characteristics
Output power	20 W
Waveband	10.55~10.63 μm
PC-1 controller	Controller component: PC-1 24 KHz
Cooling mode	Air cooling
Power	C-20A power adapter

**Table 3:** The emissivities at 10.6 µm and the true temperatures of the samples.

Samples	Rough Surface of Marble Plate (RM)	Polished Surface of Aluminum Plate (PA)	Oxidized Surface of Copper Plate (OC)	Rusty Iron Plate (RI)	Black Rubber Sheet (BR)	Paper Board (PB)
Emissivity at 10.6µm	0.844	0.102	0.362	0.239	0.964	0.871
Measured *T_s_* (*T_S_m_*, K)	301.8	300.6	301.1	301.5	301.2	301.3
Retrieved *T_s_* (*T_S_e_*, K)	301.2	301.5	301.8	300.8	301.7	301.5
Difference (K)	0.6	0.9	0.7	0.7	0.5	0.2

**Table 4:** Comparison of emissivity from two approaches.

Objects	RM	RI	BR	OC	PA
RMSE of the Spectral Emissivity	0.046	0.049	0.073	0.077	0.122
